# Food Insecurity and Affecting Factors in Households With Children During the COVID-19 Pandemic: A Cross-Sectional Study

**DOI:** 10.1017/dmp.2021.172

**Published:** 2021-06-07

**Authors:** Gizem Deniz Bulucu Büyüksoy, Aslıhan Çatıker, Kamuran Özdil

**Affiliations:** 1 Department of Nursing, Kırşehir Ahi Evran University Faculty of Health Sciences, Kırşehir, Turkey; 2 Department of Nursing, Ordu University Faculty of Health Sciences, Ordu, Turkey; 3 Aged Care Program, Nevsehir Haci Bektaş Veli University, Health Services Vocational School, Nevşehir, Turkey

**Keywords:** COVID-19, food insecurity, food supply, pandemics, socioeconomic factors

## Abstract

**Objective:**

The aim of this study was to examine the incidence of food insecurity and affecting factors in households with children in Turkey during the coronavirus disease 2019 (COVID-19) pandemic.

**Methods:**

This is a cross-sectional study. The participants were recruited by the snowball sampling method, and the data were collected by means of a link sent to their smartphones through their social media accounts. This study included 211 households with at least 1 child.

**Results:**

The study revealed that 21.8% households had food insecurity that was not at the hunger threshold. The monthly income of 80.6% of the households was below the poverty line and monthly income decreased in more than half of the households during the pandemic. Food insecurity increased 2.5 times when the households comprised workers or self-employed individuals (odds ratio [OR] = 2.529; *P* = 0.002), increased 3 times when the monthly income of the households decreased (OR = 3.131; *P* = 0.000), and increased 2 times when total monthly income of the household fell below poverty line during the pandemic (OR = 2.001; *P* = 0.049).

**Conclusions:**

It is determined that nearly half the households have food insecurity and that the pandemic poses a risk in terms of food security. We recommend that public health studies should be planned to ensure accessibility to healthy foods.

Coronavirus disease 2019 (COVID-19) has turned into a global public health emergency affecting the entire world. The World Health Organization (WHO) declared COVID-19 a pandemic on March 11, 2020. The virus, which emerged in December 2019, has affected the entire world. On September 1, 2020, a total of 25,327,098 confirmed cases of COVID-19 were reported worldwide, 848,255 of which resulted in death.^1,2^

The COVID-19 pandemic has deepened problems in many different areas, such as social, economic, and cultural, as well as health problems worldwide. Moreover, it has created new problem areas and increased inequalities. Decreased social mobility, increased unemployment, difficult access to food, and erosion of already fragile livelihoods due to curfews are possible socioeconomic consequences of the pandemic.^3,4^ In addition to the global crisis it has created, the pandemic has also caused some unprecedented humanitarian challenges, such as unemployment, economic problems, increased insecurity, and increased poverty and food insecurity.^5,6^

As has been reported, the impact and consequences of the pandemic are closely related to the social determinants of health, and the pandemic has caused more serious consequences in low-income and disadvantaged groups.^7,8^ Thus, making plans for the social determinants of health to follow effects of the pandemic and to predict its consequences is recommended.^7^ According to studies, the pandemic will predictably affect the safety of various disadvantaged groups, such as women, the elderly, children, the disabled, minorities, and asylum seekers, especially in terms of food, nutrition, and health, and it will exacerbate social and health inequalities.^9–12^ During the pandemic process, production and distribution in the food sector has been disrupted all over the world and food insecurity has increased as the purchasing power of individuals has decreased.^13^

Food insecurity is the lack of adequate access to safe and nutritious food, which is required for a healthy and active life and normal growth.^14^ Food insecurity can make it difficult to cope with pandemic as well as increase comorbidity by aggravating many chronic illnesses associated with nutrition. For example, people with a weak immune system or chronic diseases due to food insecurity were reported to be more severely at risk of catching COVID-19.^3^ However, socioeconomic problems caused by the pandemic will predictably worsen the social determinants of health, affect a large number of people, and create new and complex health problems.^7^ Decreased access to healthy food and increased food insecurity under pandemic conditions can lead to various health problems as an indirect effect of the pandemic by negatively affecting nutrition behavior, one of the health promotion initiatives.^15^

This study aims to determine the status of families with children living in Turkey in terms of food insecurity and the consumption frequency of some nutrients during the pandemic period and to examine factors associated with food insecurity.

## Research Questions

The 3 research questions asked are as follows: What is the level of food insecurity in households with children during the pandemic period? What is the consumption frequency of some essential nutrients in households during the pandemic period? What are the factors affecting food insecurity in households during the pandemic period?

## Methods

### Study Setting and Population

This is a cross-sectional study. The study comprises individuals from Turkey’s who were over 18 years old and were living in households with children. Based on the sample size (*n* = 211), the power of the study was calculated as 82% at the 0.2 effect size and 0.95 confidence level on the computer.

Participants were recruited using the snowball (chain referral) method, which is a purposive sampling method. First, potential participants were selected from individuals in the social environment of colleagues and students of the researchers, and then, more participants were reached through these participants who were their relatives, neighbors, or friends and who met the inclusion criteria of the study.

### Inclusion Criteria

Inclusion criteria for the study group are as follows: individuals who are above 18 years old, individuals who speak Turkish, individuals who are literate, individuals who do not have physical/psychiatric discomfort that prevents verbal communication, individuals who can use smartphones/can fill online surveys, individuals who live in a household with at least 1 child (under 18), and individuals who live in different living areas (rural/urban) in different provinces.

### Exclusion Criteria

Exclusion criteria for the study group are as follows: households where a person who owns any business selling food products, and households that cultivate their own land with their own tools and equipment and can produce enough products for themselves.

### Instrument

In the study, the data were collected by means of an introductory information form, a food insecurity survey for households, and a food insecurity survey for households with children.

### Introductory Information Form

In the introductory information form, the sociodemographic characteristics of the household, monthly income and monthly food expenditure in the past 5 months, economic changes (dismissal from work, decrease in monthly income, increase in food expenditure, etc.), consumption frequency of some essential nutrients (milk and dairy products, red meat and meat products, eggs, bread, and bakery foods, cereals and dried legumes, fruits and vegetables, chicken, fish) and health problems of the household in the past 5 months were questioned.

### Food Insecurity Survey

Food insecurity status of the households were measured using an 8-item survey for the accessibility of households to food and a 6-item food insecurity survey for households with children, developed by Eştürk (2013) using the US Household Food Security Survey Module. The items in the surveys comprise questions about the household and children’s access to food as well as their nutritional behavior. Answers are in the form of often (2 points), sometimes (1 point), never (0 point), yes (1 point), or no (0 point). After data collection, the total score was calculated, and the food security status of the individuals was evaluated according to the total score calculated. The survey evaluated food security (0 point), food security at risk (1-2 points), food insecurity that is not at hunger limit (3-7 points), food insecurity with moderate hunger (8-12 points), and food insecurity with severe hunger (13 points and above).^16,17^ This survey covers the amount of food consumed by households, their quality, and perception of food security. The survey reveals the families’ food consumption behaviors, experiences, and difficulties in accessing food. The questions address the problems in accessing food caused by a lack of money or other resources. This survey was used to determine the hunger anxiety and food access difficulties of adults and children.^17^

### Data Collection

The research data were collected by an online survey that consisted of software or survey instruments used within the social media and a link sent to participants’ smartphones through their social media accounts. Before application, the survey forms were pre-applied to 10 people outside the research sample, incomprehensible parts were corrected, a few questions were shortened for the ease of use of the online questionnaire, and the survey was finalized. The online forms were designed in a way that participants can fill them out only once. In the study, before the survey forms, information about the purpose of the study and a consent option was added for participants to confirm their participation. The participants were included in the study and were able to answer the research questions only after clicking the “I agree” option. The data were collected between August 16, 2020, and September 2, 2020.

### Data Analysis

The descriptive statistics of the data were evaluated with numbers and percentages. Conformity of the distributions of the examined variables with normal distribution was analyzed by the Shapiro-Wilk test. A multivariate linear regression analysis was performed to determine variables that are predictors of food insecurity. In the regression analysis, the dependent variable is the food insecurity scale score, the independent variables are the occupation of home workers, the decrease in income entering the house in the pandemic, the change in the amount allocated for food, the monthly income of the household, and the layoffs in the pandemic. Significance level was accepted as *P* < 0.05.

### Ethical Considerations

Before starting the research, the approval of the Scientific Research Board of the Ministry of Health (2020-08-11T10_01_25) and the ethics committee approval (08-13-2020/2020.16.196) from Nevşehir Hacı Bektaş Veli University were obtained. In addition, the purpose, plan, and duration of the study were explained to the participants and their consents were obtained.

## Results

In the study, one individual from each household was interviewed. The average age of the interviewed individuals is 33.81 ± 7.76, and 76.8% of them are the mothers in the family. In addition, 82.5% of the individuals are women, 91.5% are married, 40.3% are university graduates, 80.6% have social security, and 46.0% are housewives (Table 1).

**Table 1.** Characteristics of the individuals interviewed in the household (*n* = 211)

Characteristics	*n*	%
Age, y (mean ± SD) 33.81 ± 7.76		
Family role		
Mother	162	76.8
Father	36	17.1
Other	13	6.2
Gender		
Women	174	82.5
Men	37	17.5
Marital status		
Married	193	91.5
Single	18	8.5
Educational status		
Graduate	18	8.5
Undergraduate	85	40.3
High school	52	24.6
Middle school	40	19.0
Primary school	16	7.6
Social Security		
Presence	170	80.6
Absence	41	19.4
Occupation		
Civil servant	61	28.9
Tradesman	15	7.1
Self-employment	13	6.2
Worker	17	8.1
Housewife	97	46.0
Student	8	3.8

Three or 4 individuals are present in 75.4% of the households, and 3 or more children are present in 13.7% of the households. Only one person working individually is present in 65.4% of the households, while at least 1 unemployed person is there in 58.8% of the households. In 48.5% of the households, the employed people are officer/tradesmen. During the pandemic period, one person was dismissed from work in 15.6% of the households, a decrease occurred in monthly household income in 55.5% of the households, and the share spent on food increased in 48.3% of the households. In the past 6 months, the monthly income is below the poverty line in 80.6% of the households. In 31.8% of the households, at least one health problem was experienced in the past 6 months. The health problems experienced were common cold (38.9%), chronic disease (26.9%), COVID-19 (22.3%), and psychological problems (16.4%) (Table 2).

**Table 2.** Descriptive characteristics of the household (*n* = 211)

Characteristics	*n*	%
Number of individuals living in the household		
Three or four	159	75.4
Five and more	52	24.6
Access to clean drinking water		
Yes	168	79.6
Sometimes	9	4.3
No	34	16.1
Number of children living in the household		
1-2	182	86.3
3-4	29	13.7
Number of employee in the household		
None	9	4.3
1	138	65.4
2-10	64	30.3
Number of unemployed in the household		
None	87	41.2
1	80	37.9
2-10	44	20.9
Occupation of employees in the household (*n* = 202)		
Civil servant/tradesman	98	48.5
Self-employment/worker	104	51.5
Has anyone been laid off in the pandemic?		
Yes	33	15.6
No	178	84.4
Has there been a decrease in household monthly income during the pandemic process?		
Yes	117	55.5
No	94	44.5
Change in money spent on food in the pandemic		
Increased	102	48.3
Not changed	56	26.5
Decreased	53	25.1
Household monthly income		
Above the poverty line	41	19.4
Below the poverty line	170	80.6
Has there been any health problem in the past 6 months?		
Yes	67	31.8
No	144	68.2
Experienced health problems more than 1 answer (*n* = 67) ^a^		
Common cold	26	38.9
Chronic illness ^a^	18	26.9
COVID-19	15	22.3
Psychological problems	11	16.4
Waist-leg-muscle pain	7	10.4
Excessive weight gain	4	6.0

a Type II Diabetes and Hypertension Attack, Cardiovascular Disease

**Figure 1.**
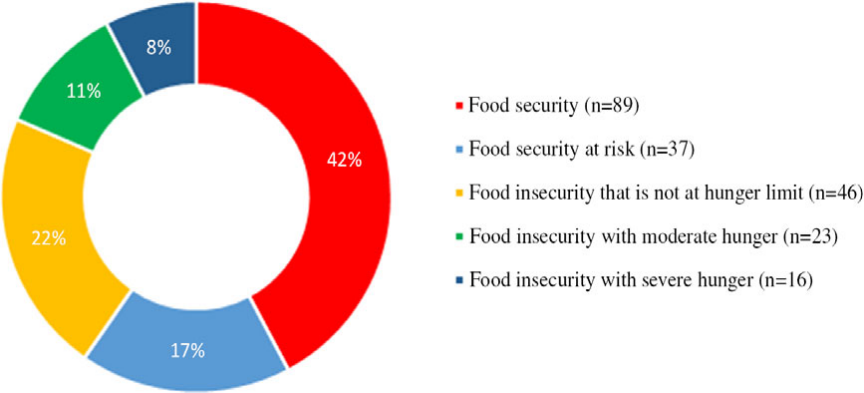
Distribution of food insecurity.

**Figure 2.**
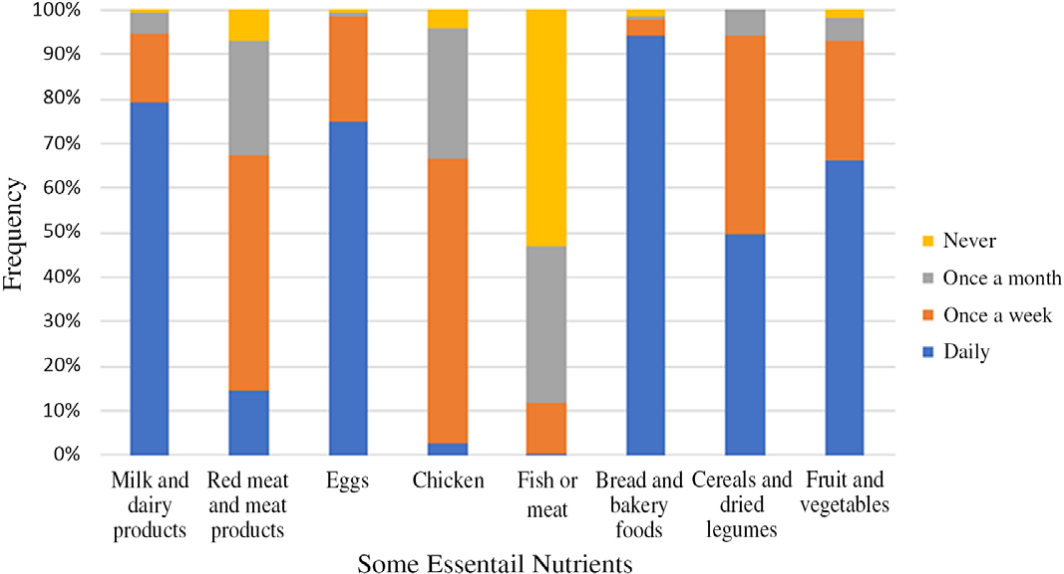
Distribution of households by type and frequency of some essential nutritients consumption in the last six months.

In the study, 42.2% of the households had food security. However, food security was at risk in 17.5% of the households; food insecurity that was not at hunger limit was there in 21.8% of the households, food insecurity with moderate hunger was there in 10.9% of the households, and food insecurity with severe hunger was there in 7.6% of the households (Figure 1). In addition, 79.1% of the households stated that they consumed milk and dairy products, 74.9% of the households consumed eggs, 94.3% of the households consumed bread and bakery foods, 49.8% of the households consumed cereals and dried legumes, 66.4% of the households consumed fruit and vegetables daily during the past 6 months. Furthermore, 52.6% of the households consumed red meat and meat products once a week, 64.0% of the households consumed chicken once a week, and 53.1% of the households never consumed fish or meat (Figure 2).

According to the multiple linear regression analysis performed in the study, the profession of employees in the household, the change in the household income level in the pandemic process, the change in the amount spent on food in the households in the pandemic process, the monthly income, and presence of an individual in the household who was dismissed from work during the pandemic period explains 24% of food insecurity (R^2^ = 0.239; *P* < 0.001). Accordingly, food insecurity increased 2.5 times when the occupation of the household employees was worker or self-employed (*P* < 0.01), increased 3.1 times when the household had a decrease in the monthly income (*P* < 0.001), and increased 2.0 times when total monthly income of the household fell below poverty line during the pandemic period (*P* < 0.05) (Table 3).

**Table 3.** Variables predicting food insecurity multivariate regression analysis (*n* = 211)

Variables predicting food insecurity	B	SE	*B*	t	*P*-Value	95% CI	
						Lower	Upper
Occupation of employees in the household	2.529	0.812	0.215	3.116	**0.002**	0.929	4.129
Has there been a decrease in household monthly income during the pandemic process?	3.131	0.837	0.266	3.743	**0.000**	1.482	4.781
Change in money spent on food in the pandemic	0.203	0.494	0.029	0.410	0.682	-0.771	1.177
Household monthly income	2.001	1.010	0.135	1.982	**0.049**	0.010	3.993
Has anyone been laid off in the pandemic?	0.366	1.159	0.023	0.316	0.752	-1.919	2.651

*Note:* Values in boldface are considered significant. *R* ^2^ = 0.239; *P* < 0.001.

## Discussion

It is predicted that social, cultural, and economic changes that occur during the pandemic process will affect the nutritional status of individuals and societies, their access to food and food security. The results of this research are important in terms of determining food insecurity and the factors affecting it during the pandemic process.

We determined that most of the households included in the study comprised 3 or 4 people, and 1 or 2 children were present in more than half of the households. Moreover, only one person was employed in more than half of the households, half of the employees were workers or self-employed, and at least one person in every 2 households was unemployed. Thus, we can conclude that households mostly had socioeconomic difficulties. However, in addition to the existing situation of the households, the pandemic caused extra problems. When the effect of pandemic conditions on households was examined herein, we found that at least one individual was dismissed from work in 1 of every 6 families due to the pandemic and the monthly income decreased in more than half of the households (Table 2).

A study conducted in Mexico stated that food security in the households with children gradually decreased from 38.9% in 2018 to 24.9% in June 2020.^18^ Similarly, a study conducted in the United States determined that the number of families that were reported to have a very low food security increased by 20% during the pandemic.^19^ In Bangladesh, the number of families with food insecurity at any level was reported to have increased by 51.7% during the pandemic.^20^ In Ethiopia, indicators that measure food security were found to have significantly deteriorated compared with the period just before the epidemic.^21^ A report by the UK Food Foundation reported that the number of adults with no food security quadrupled in the United Kingdom during the pandemic and that households with a disabled individual or child are more vulnerable.^4^ In this study, the frequency of household food insecurity is 40.3% (Figure 1). A study examining unemployment and food insecurity identified that 40.5% of those who were employed before the pandemic in the United States were unemployed during the pandemic outbreak; furthermore, 31% of those who were unemployed experienced food insecurity, and 33% ate less food due to economic losses.^22^ Similarly, in this study, in 15.6% of the households, at least 1 individual was dismissed during the pandemic process, and at least 1 person in 58.8% of the households was unemployed, and 25.1% of the households stated that the share allocated for food during the pandemic process decreased (Table 2). However, in this study, food insecurity increased 2.5 times when the occupation of the household member was a worker or self-employed, increased 3.1 times when the household had a decrease in the monthly income, and increased 2.0 times when the total monthly income of the household fell below the poverty line during the pandemic period (Table 3) (*P* < 0.005).

During the pandemic period, money spent on food increased in nearly half of the researched households, and conversely, money spent on food decreased in a quarter of the households (Table 2). Poorer segments of the society allocate more of their monthly income to food compared with the wealthy.^23^ In this survey, despite the decrease in unemployment and monthly income, the increase in the share of food suggests that households try to maintain their food security at a certain point by increasing the amount they allocate to food. The fact that the prevalence of food insecurity was found in less than half the households in the study may be related to this. One study explained that food security in households was not worrying because families used their savings to support their food consumption. However, it was emphasized that this support has no chance of lasting long.^21^ In addition, in Turkey, the consumer price index in August increased by 2.3% compared with the previous month.^24^ Accordingly, even if same food items were purchased in the same amount in households, the money paid for them might have increased. This might have increased the amount spent on food. In a study where the food security in 45 countries during the pandemic was examined, a threat to access to food for the poor was present as a result of loss of income resulting from the pandemic restrictions of social life, cessation of trade, food inflation, and depreciation of currency; food insecurity may be experienced higher in countries that depend on imports in food supply, such as Turkey.^25^

Herein, we determined that a health problem was experienced in 31.8% of the households in the past 6 months, and the health problem experienced was mostly a cold or chronic disease (Table 2). The fact that the most common health problem was a cold on days when the air temperature was high suggested that individuals’ immune system resistance was low. During the pandemic period in the past 6 months, the most consumed food in the households was bread daily, and the least consumed foods were fish, chicken, red meat, and meat products (Figure 2). The health status of individuals was negatively affected as animal foods were consumed less in the households, one-fifth of households did not consume milk, dairy products, and eggs daily, and nutrition was mostly based on bread consumption. Food insecurity can lead to a worse health status as it affects the nutritional value and quantity of selected foods.^26^ For example, one study determined that approximately one-third of families purchased high-calorie snacks and desserts more often during the pandemic period.^19^ In addition, the aggravation of chronic diseases in some individuals during the pandemic process can be explained by the disruption of the individuals’ access to health services during the pandemic, the decrease in active life opportunities, and malnutrition. Strict quarantine conditions were reported to create adverse metabolic conditions and increase the risk of diabetes, osteoporosis, cancer, and cardiovascular diseases due to reduced physical activity.^27^

## Conclusions

The study results emphasize the impact of COVID-19 pandemic conditions on socioeconomic conditions of households as well as prevalence of food insecurity in households and affecting factors. The results are consistent with previous studies that showed how socioeconomic conditions affect food insecurity of households.

Considering the social background of food insecurity, which seriously affects low and middle-income households, the problem clearly cannot be solved by individual efforts. Global interventions are needed to reduce food insecurity and its effects to combat this problem. On the other hand, local interventions are of great importance for the fast and effective solution of food insecurity. Thus, public health professionals who could follow a healthy individual and provide services in primary care need to be aware of food insecurity. Moreover, regularly monitoring and reporting the frequency of household food insecurity is important, and supportive public health policies need to be developed to ensure access to food for particularly disadvantaged groups.

### Limitations of the Study

Because face-to-face interviews were not possible with individuals due to pandemic conditions, the individuals constituting the sample of the study could not be homogeneously and randomly selected from the regions, and only individuals with known contact information could be reached. Therefore, the results of the study can only be generalized to this sample group. The heterogeneity of demographic variables, such as the region where the sample group lives, education level, and income level is another limitation in terms of generalization of the study results.
